# Comparative Accuracy and Reliability of Three Electronic Apex Locators in Determining the Apical Constriction of Molar Canals: A Micro-CT Evaluation

**DOI:** 10.3390/jcm13175199

**Published:** 2024-09-02

**Authors:** Reem M. Barakat, Rahaf A. Almohareb, Arwa O. Alharbi, Asma Alhazmi, Reem Alomar

**Affiliations:** 1 Dental Clinics Department, King Abdullah bin Abdulaziz University Hospital, Princess Nourah Bint Abdulrahman University, P.O. Box 84428, Riyadh 11671, Saudi Arabia; rmbarakat@pnu.edu.sa; 2 Department of Clinical Dental Sciences, College of Dentistry, Princess Nourah Bint Abdulrahman University, P.O. Box 84428, Riyadh 11671, Saudi Arabia; 3 Dental Intern, College of Dentistry, Princess Nourah Bint Abdulrahman University, P.O. Box 84428, Riyadh 1167, Saudi Arabia; arwaharbi75@hotmail.com (A.O.A.); asmaakalhazmi@gmail.com (A.A.); reemalomar35@gmail.com (R.A.)

**Keywords:** apical constriction, electronic apex locator, micro-CT imaging, root canal treatment, working length

## Abstract

**Background:** Determining the correct apical limit for root canal treatment is essential for its success. This study evaluates the accuracy of three electronic apex locators (EALs) in locating the apical constriction (AC) in molar canals. **Methods:** Forty extracted human mandibular molars were scanned using micro-CT, and endodontic access cavities were created. Teeth were mounted in alginate, and three EALs—Root ZX-mini, Root ZX-II, and Sirona integrated apex locator—were used to measure the canal working length in dry canals and with EDTA gel. Micro-CT scans were performed with files in place, and the distance from the AC was calculated. Measurements within 0.1–0.5 mm were categorized as ‘close’. Those extending beyond towards the major foramen were categorized as ‘beyond’, otherwise they were classified as ‘far’. Data analysis was conducted with a level of significance set at 5%. **Results:** Most readings for all EALs were in the ‘close’ category, with significant differences between devices (*p* < 0.0001). Root ZX-mini and Root ZX-II had 74.4% and 72.5% ‘close’ readings, respectively, versus 51% for Sirona integrated. Accuracy did not differ significantly between dry and EDTA-treated canals (*p* = 0.306). All EALs demonstrated excellent operator reliability (ICC 0.996–1.00). **Conclusions:** All EALs accurately determined AC, unaffected by lubricants. However, Root ZX-mini and Root ZX-II outperformed Sirona integrated. All EALs showed consistent reliability.

## 1. Introduction

The outcome of root canal therapy is strongly dependent on determining an appropriate apical limit for instrumentation and root canal filling [1]. Incorrectly estimating working lengths (WLs) might result in inadequate disinfection or injury to periapical tissues, leading to the absence of periapical healing [2].

The complexity of the apical part of the root canal system presents challenges in selecting the appropriate endpoint for instrumentation and filling. Two key landmarks in this area are the apical constriction (AC) and the apical foramen (AF). Accurate identification of these landmarks is crucial for effective treatment, ensuring that cleaning and filling procedures completely remove infected tissue while staying within the root canal. This helps prevent damage to the surrounding periapical tissues, which can lead to inflammation, pain, and delayed healing.

The AC is the narrowest part of the root canal near the apex, but its precise location can vary among different roots. In contrast, the AF is the natural anatomical opening at the apex where the nerves and blood vessels enter and exit the tooth. This opening may not always align exactly with the anatomical apex and can be located up to 3 mm away [3,4].

The AC is often preferred as a landmark because it can help achieve a natural seal, though it may be affected by pathology and is not visible on radiographs. The AF, while more consistent, can be less precise and poses higher risks of over-instrumentation and debris extrusion [5]. Therefore, determining the working length (WL) requires a combination of methods, including radiographs, electronic apex locators (EALs), tactile sensation, the paper point technique, and a thorough understanding of root canal anatomy [5,6].

The idea of electronically measuring root length was first proposed by L. E. Custer in 1918 [7], but it was not until 1962 that Sunada developed a device using direct current to determine working length (WL) [8]. Sunada’s design was based on Suzuki’s 1942 discovery that the electrical resistance between the periodontal ligament and oral mucosa is consistently 6.5 kiloohms. The second generation, introduced in 1971, used alternating current to measure impedance but still showed limited accuracy especially in the presence of electrolytes. The third generation of EALs launched in 1994 by Kobayashi with the Root ZX (J. Morita; Kyoto, Japan) which was able to overcome these drawbacks by measuring impedance at two different frequencies [9]. Over the decades, digital technology and applying algorithms to improve impedance measurement significantly advanced EAL devices, resulting in more accurate, user-friendly, and integrated models [10].

In recent years, EALs have become integral to WL determination, recommended by the European Society of Endodontology (ESE) and American Association of Endodontology (AAE) alongside radiographs [11,12]. These devices operate by measuring electrical impedance and capacitance within the root canal to calculate the file’s position accurately. Development of EALs has passed through many generations. While the first two generations are deemed obsolete due to their inaccuracy, the later generations of EALs have proved to be highly accurate in measuring the WL [13]. EALs not only mitigate the drawbacks of relying solely on radiographs—such as radiation exposure, time consumption, and the interpretation challenges posed by two-dimensional images [14,15,16]—but they are also valuable tools for locating the AC [5,17]. However, many variables such as metallic restorations, canal content, and anatomical complexities might impair their accuracy [18,19,20].

EALs are available as standalone devices or integrated into endodontic motors or dental chairs. One of the most notable standalone EALs is the Root ZX (J. Morita; Kyoto, Japan). Extensively studied since its introduction, the Root ZX has demonstrated excellent performance, establishing it as the gold standard among EALs [21,22]. The device is a third-generation EAL which determines the file’s position inside the canal by calculating the impedance ratio at two frequencies: 0.4 and 8 kHz [23]. This impedance ratio adopts certain values as the file approaches the apical constriction or foramen [5].

The original Root ZX was later replaced by two updated models: the Root ZX-II and the Root ZX-mini. These devices operate on the same principle as the original Root ZX but offer additional features such as the ability to integrate with an endodontic motor and a compact design (Root ZX-mini) [22,23].

The Dentsply Sirona Intego Pro dental chairs, manufactured by Dentsply Sirona (Sirona, Charlotte, NC, USA), incorporate an integrated EAL to streamline workflow efficiency during root canal therapy. According to the manufacturer’s manual, this device is designed to determine the working length by evaluating electrical impedance between the file and a mucosal electrode, capable of detecting what the manual refers to as the apical constriction or “physiological apex”. However, the device’s accuracy may be affected by electromagnetic fields, which could lead to measurement errors [24,25].

Micro-computed tomography (micro-CT) has emerged as a precise, non-invasive tool for assessing root canal anatomy. Unlike conventional 2D radiographs, this technology allows for clear visualization of both the AC and AF without altering or damaging the tooth. It enables accurate measurement of the distances between EAL readings and the actual locations of the AC and AF, making it particularly useful for assessing the accuracy of EALs, especially in detecting the AC [26,27,28]. Despite its reliability, few studies have applied micro-CT to assess EAL accuracy comprehensively.

Research on EALs has predominantly focused on straight, single-rooted teeth [29], although most root canal treatments involve multi-rooted molars [30]. The limited studies conducted on curved molar canals mainly involved Root ZX [31]. Moreover, no studies to date have examined the performance of chair-integrated EALs.

Therefore, this study aims to compare the performance of three EALs—the Root ZX-II, Root ZX-mini, and the Sirona Intego Pro integrated Apex Locator (Sirona integrated)—in locating the apical constriction (AC) in molar canals under both dry and wet conditions. The null hypothesis posits no difference among these three EALs.

## 2. Materials and Methods

The Institutional Review Board of Princess Nourah Bint Abdulrahman University granted exemption for this ex vivo study (IRB #23-0631).

### 2.1. Sample Selection

Sample size calculation was conducted using G*Power 3.1 software (Heinrich-Heine-Universität, Düsseldorf, Germany). At a level of significance of α = 5%, an estimated standard deviation of 0.05, and a power of 95%, the total sample size was determined to be 60 canals. Forty lower first or second mandibular molars extracted due to periodontal issues were included after obtaining informed consent from patients or their legal guardians. Exclusion criteria comprised teeth with fused canals, an immature apex, calcified canals, previous endodontic treatment, caries, cracks, or any indication of internal or external root resorption, and teeth with excessively long (>25 mm) or short (<18 mm) roots. This ensured a homogeneous sample, as variations in tooth length have been shown to impact the accuracy of EALs [32]. In cases of joining root canals with a single foramen, only one root canal was included.

### 2.2. Sample Preparation

**Figure 1:**
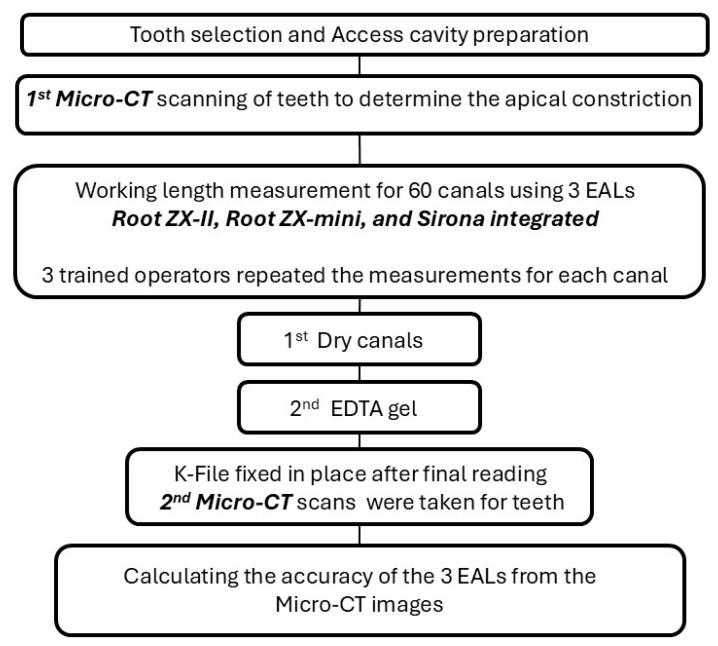
Schematic representation of the experimental workflow.

The external root surfaces were scaled with ultrasonic instruments and washed with distilled water to remove calculus or soft tissue. Teeth were stored in phosphate-buffer saline until used. A flowchart describing the subsequent experimental protocol can be seen in Figure 1.

An endodontic access cavity was prepared in each tooth using a #14 round diamond bur and finished with an Endo Z bur (Dentsply Maillefer, Ballaigues, Switzerland) under water spray, and orifices were enlarged using SX Protaper Gold orifice openers (Dentsply, Sirona, Charlotte, NC, USA). The buccal and lingual cusps were flattened with a diamond disk and used as coronal reference positions for the k-file. Each tooth was placed in a container filled with alginate to replicate the conduction of electricity and simulate an oral environment [33,34]. The procedure involved mixing the alginate (Zetalgin, Zhermack SpA, Badia Polesine, Italy) according to the manufacturer’s instructions. After pouring it into the container, the root of the tooth was immediately inserted in the container center until the alginate reached the level of its CEJ. The tooth was held in place until the alginate set. Measurements were conducted within the span of 1 h to ensure that the alginate maintained adequate moisture.

### 2.3. Measurement of the Working Length (WL)

**Figure 2:**
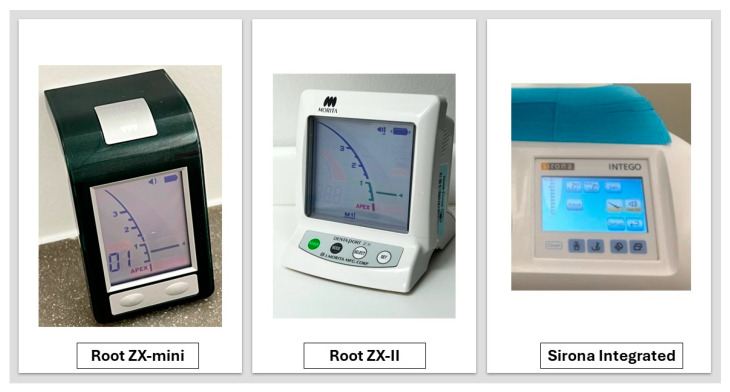
Image of the three EALs examined in this study.

Size 10 K-files (Kerr Dental, CA, USA) were used to negotiate the canal till its working length (WL). Canals that were non-negotiable to full WL or experienced instrument fracture were excluded. Three EALs were used: Root ZX-II, Root ZX-mini, and Sirona integrated following manufacturer instructions (Figure 2). The lip clip was placed into the alginate, and the wire from the apex locator was connected to the file. Measurements were taken twice: the first time in dried canals, then with Ethylenediaminetetraacetic acid (EDTA) gel (MD-Chelcream, Meta Biomed, Osongsaengmyeong, Republic of Korea) placed on the tip of the file. The file was inserted into the canal until the EAL reading indicated the “apex” red line, then gently withdrawn to the specified green line (final green bar on the Root ZX-II and Root ZX-mini devices). Three trained operators repeated the WL measurements. The operators were blinded to their own and each other’s measurements. All measurements were recorded using a high-precision digital caliper (Fowler IP 54, Newton, MA, USA).

### 2.4. Micro-CT Scanning and Determining Distance from Apical Constriction

Each tooth was scanned twice using a Bruker SkyScan 1172 high-resolution micro-CT scanner (Bruker SkyScan, Kontich, Belgium). The following standardized configurations were applied: 99 kV voltage, 100 μA anode current, 316 ms exposure time, 17 μm image pixel size, 0.6° rotation step for a full 360° rotation, frame averaging of 4 to enhance the signal-to-noise ratio, random movement of 8 to minimize ring artifacts, and a Cu+Al filter to reduce beam hardening effects.

After scanning, image reconstruction was performed using N-Recon version 1.6.9.4 (Bruker SkyScan, Kontich, Belgium). Parameters for optimal image quality, especially with the presence of the metal K-files were adjusted for the first and second scans. These included the following: ring artifact reduction set to 9 for background image non-uniformity, 40% beam hardening compensation for the first scan (without the file) and 23% for the second scan (with the file), and smoothing with a Gaussian kernel set to 2, while contrast limits ranged from 0.0 to 0.12 when the file was present and from 0.0 to 0.05 without the file [35]. The reconstructed images were analyzed using the DataViewer program version 1.5.6.2 (Bruker SkyScan, Kontich, Belgium). Color filter and inverse imaging were also compared during analysis of the images with files. A tooth containing a calibrated cavity was scanned following the same protocol and parameters to ensure precise distance measurement.

### 2.5. First Scan—Pre-WL Measurement

**Figure 3:**
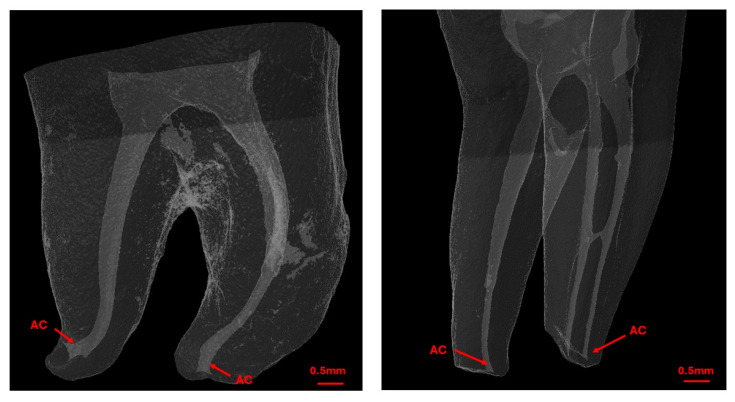
Micro-CT reconstructions of the tooth showing the external and internal anatomy of the root canal system with AC highlighting the position of the apical constriction.

Prior to determining the WL of root canals, the first scan captured detailed anatomical data. A single operator identified the apical constriction (AC) as the most apical zone with the smallest cross-sectional area that extends at least 0.1 mm (Figure 3). The most apical position of the AC was marked and used as a reference point for the subsequent EAL measurements. During this scan, the distance between the AC and major foramen (AF) was recorded for each canal.

### 2.6. Second Scan—Post-WL Measurement

**Figure 4:**
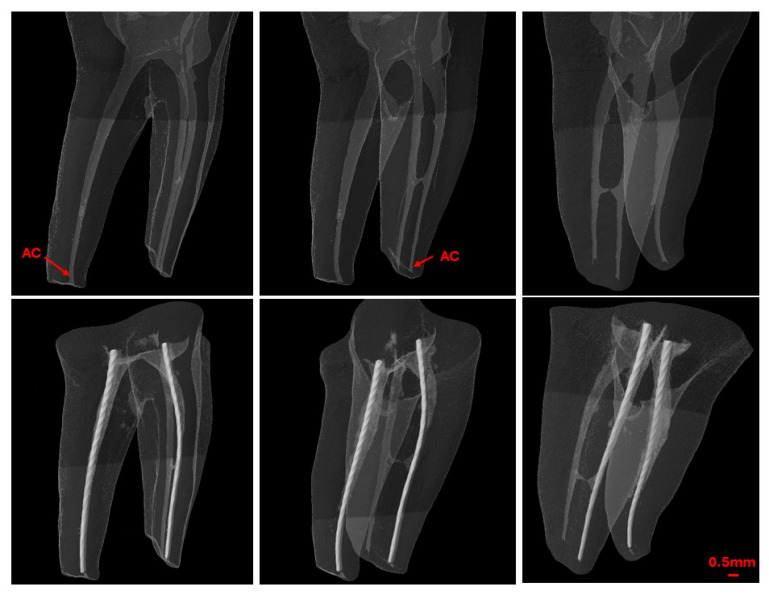
Pre- and post-working length measurement micro-CT scans.

After establishing the WL using EAL, a second scan was conducted with the endodontic file fixed within the canal (Figure 4). The pre- and post-scan datasets were co-registered, and the distance between the tip of the endodontic file and the previously identified AC was calculated and recorded as positive or negative values relative to the AC.

For clarity and accuracy assessment, the recorded measurements falling within 0.1–0.5 mm from the AC were categorized as ‘close’. Measurements extending beyond the tolerance level towards the major foramen were categorized as ‘beyond’, otherwise they were classified as ‘far’.

### 2.7. Data Analysis

SPSS software version 22 (SPSS Inc., Chicago, IL, USA) was used for data analysis. Descriptive statistics (means, standard deviations, and confidence intervals) were calculated and compared. The Shapiro–Wilk test was used to check data normality, and, accordingly, it was analyzed using a two-way ANOVA test. The Chi-square test and percentage accuracy were reported. Statistical significance was set at α ≤ 0.05. Intraclass correlation coefficient (ICC) and coefficient of repeatability assessed EAL reading reliability [36].

## 3. Results

### 3.1. Accuracy of Electronic Apex Locators (EALs)

**Figure 5:**
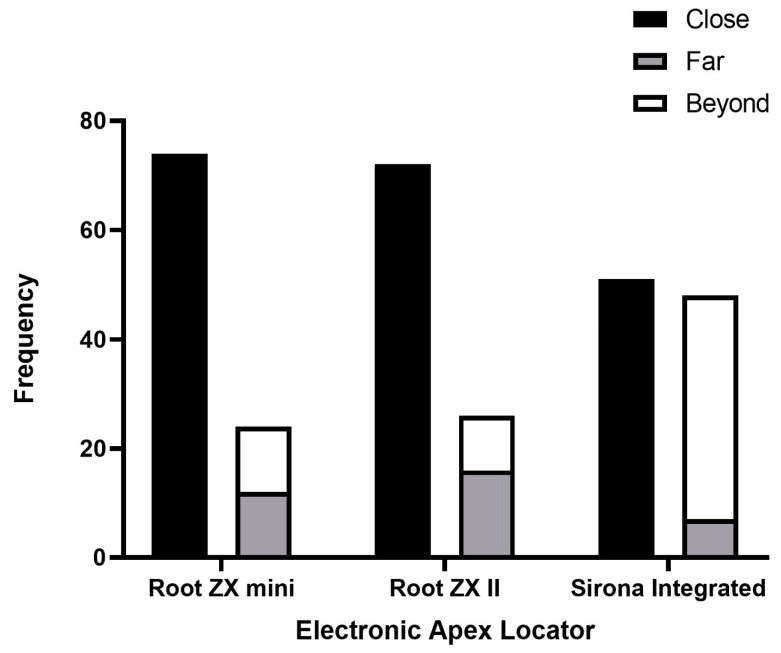
Accuracy of three EALs in detecting the apical constriction within a tolerance of 0.5 mm.

The majority of measurements from all EALs predominantly fell within the ‘close’ category, indicating a high level of accuracy in determining the AC. However, there was a statistically significant difference observed between the devices (*p* < 0.0001). Specifically, Root ZX-mini and Root ZX-II demonstrated 74.4% and 72.5% of measurements in the ‘close’ category, respectively, whereas the Sirona Integrated device showed only 51% (Figure 5).

Comparing measurements taken in dry canals versus those where EDTA gel was used, the mean distance from the apical constriction recorded via the EALs was slightly lower in the dry group. However, this difference did not reach statistical significance (Table 1 and Table 2).

### 3.2. Deviation from Apical Constriction

**Figure 6:**
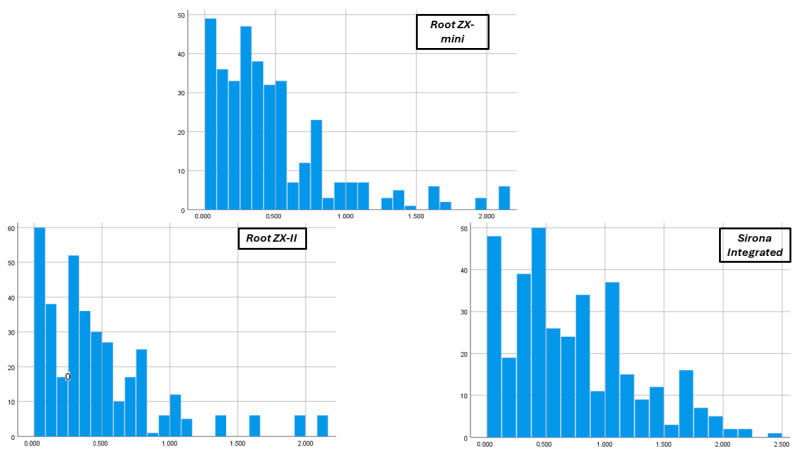
Distribution of absolute deviation values from the apical constriction.

Root ZX-mini and Root ZX-II exhibited comparable absolute values of deviation from the apical constriction, as depicted in Figure 6. Notably, Root ZX-II had a slightly lower percentage of “beyond” readings (10.8%) compared to Root ZX-mini (12.5%), although not statistically significant (Figure 6).

This figure displays the absolute deviation values from the apical constriction. The horizontal axis shows the measured distance between the tip of the file and the apical constriction, while the vertical axis represents the frequency of these measurements.

### 3.3. Reliability of EAL Readings

All three EALs demonstrated high reliability, with intraclass correlation coefficients (ICCs) ranging between 0.996 and 1.00. Root ZX-II achieved the highest ICC value among the devices tested. The repeatability coefficients, indicative of measurement consistency, were calculated as 0.12 mm for Root ZX-mini, 0.09 mm for Root ZX-II, and 0.15 mm for the Sirona Integrated device. These coefficients are considered acceptable, with over 95% of repeated measurements showing differences below the respective coefficients. No significant difference in repeatability coefficients was found among the three EALs.

## 4. Discussion

This study compared the accuracy of two standalone electronic apex locators (EALs), Root ZX-II and Root ZX-mini, against the Sirona integrated EAL which is integrated within the Sirona Intego Pro dental chair. The findings revealed that both Root ZX-II and Root ZX-mini consistently outperformed the integrated device in accurately detecting the apical constriction within a 0.5 mm tolerance. These results are consistent with prior research comparing these EALs to devices of the same or later generations [35,37,38].

Given the complexity of the root canal system, a chemo-mechanical approach is essential for successful treatment. This approach combines mechanical instrumentation of the canal with the use of irrigation fluids that can remove both organic and inorganic tissue, eradicate bacterial biofilm, and serve as lubricants. This combination is critical for thoroughly cleaning the canal and ensuring effective bacterial elimination [39].

EDTA is a chelator agent which binds to Ca^2+^ ions enabling removal of inorganic tissue and debris, otherwise known as the smear layer. In addition to being the most effective smear layer remover, 17% EDTA also promotes the release of growth factors in regenerative endodontics [40]. EDTA is used alongside sodium hypochlorite (NaOCl): the most common root canal irrigant. NaOCl is highly valued not only for its strong antibacterial properties but also for its excellent ability to dissolve tissue, effectively removing vital and necrotic pulp tissue during root canal treatment [39,41].

During chemo-mechanical preparation of root canals, lubricants like EDTA gel are commonly used to enhance cutting efficiency and reduce stress on endodontic files [42]. Manufacturers of rotary files recommend that a gel lubricant is placed on each file before insertion into the root canal, while some dentists also suggest using it at the beginning of the procedure with hand files [41,43].

This study investigated whether EDTA gel affects EAL performance and found that its presence did not diminish the accuracy of any of the three EALs studied. This can be attributed to EALs’ impedance-based calculations, which mitigate the influence of electrolytes in the canal [5]. One study compared gel- and solution-type irrigants and found that the gel type was more reliable in determining the WL of perforated apices. This was explained by the three-dimensional structure of gel, the concentration of ions, and the water content which complicates its electro-conductive behavior [44]. A more recent study, however, found that irrespective of their electrical conductivity, root canal irrigants did not adversely affect the accuracy of third-generation EALs [45]. However, conflicting findings exist in the literature regarding the impact of moisture-enhancing agents like EDTA on EAL accuracy. Some studies indicate that EDTA does not significantly affect accuracy, while others have shown that it reduces accuracy compared to NaOCl. This discrepancy may be due to pulpal tissue remnants in the root canal, which can decrease EAL accuracy in clinical settings. By dissolving these tissues, NaOCl improves EAL performance [46,47,48].

The differing results may also stem from variations in study design, methodologies, and measurement tools. None of the cited studies used micro-CT to evaluate the accuracy of EALs in locating the apical constriction (AC). In this study, micro-CT was selected for its non-destructive nature and its ability to precisely identify the AC. This method surpassed traditional longitudinal root sectioning, known for its limitations in accurately defining anatomical landmarks [26].

In the present study, the Sirona integrated EAL proved to be less accurate compared to both the Root ZX-II and Root ZX-mini, achieving a ‘close’ reading in only 51% of the measurements. Additionally, it was more prone to overestimating the working length, with 41% of the readings extending ‘beyond’ the apical constriction. This may be attributed to differences in the specific algorithms used by the manufacturer [35]. Another possible explanation is that the integration of this device within the dental chair could make it more susceptible to electrical interference, thereby compromising its accuracy.

Root ZX-II and Root ZX-mini demonstrated accurate apical constriction detection in 72.4% and 74.5% of cases, respectively. Variability in accuracy levels for the Root ZX-II (62–97%) and Root ZX-mini (56.2–95%) has been reported across different studies, likely due to differences in study methodologies [37,49,50,51]. This study uniquely examined the accuracy of the Root ZX-mini and Root ZX-II in molar canals using micro-CT, revealing comparable results in absolute deviation from the apical constriction. While no significant difference was found in overall accuracy, the Root ZX-II demonstrated a lower percentage (10.8%) of ‘beyond’ readings compared to the Root ZX-mini (12.5%), supporting previous research that suggests the Root ZX-II is more effective in avoiding overestimation of working length [52].

All three EALs exhibited high repeatability, consistent with previous findings [53]. Despite no statistically significant differences observed among devices, Root ZX-II displayed a lower repeatability coefficient, further highlighting its reliability in clinical use.

This study emphasizes the critical role of choosing reliable EALs for accurate working length determination during root canal procedures. Variations in device performance underscore the importance of practitioners selecting apex locators that consistently deliver dependable results. EALs are an upfront investment for dental practices, but they provide substantial cost savings in the long run. They are easy to use and enhance efficiency by minimizing the need for multiple radiographs, reducing procedure times, and allowing repeated use without radiation exposure [54]. Furthermore, they are effective in detecting perforations, resorptions, and root fractures [55,56]. While integrated EALs are advantageous in terms of ergonomics, offering space and time efficiency, standalone devices provide benefits in portability, simplicity, and, as demonstrated in the current results, precision [10].

The present study provides valuable insights into the comparative accuracy and reliability of different EALs, highlighting Root ZX-II and Root ZX-mini as preferable choices for precise working length determination in endodontic practice. All three EALs demonstrated high reliability, as evidenced by their consistent repeatability. This study also found that EDTA gel does not affect the accuracy of these devices, reassuring clinicians that using lubricants like EDTA during chemo-mechanical preparation is unlikely to compromise their performance.

Limitations, such as the ex vivo nature of micro-CT due to radiation concerns, restricted this study’s ability to fully replicate clinical conditions. Future research should aim to confirm these findings across a broader range of clinical scenarios to further improve treatment outcomes. This should explore how EALs perform in different clinical scenarios, including the presence of pulpal tissue remnants, calcifications, and complex anatomical features [47,57].

## 5. Conclusions

Under the conditions of this ex vivo study, the Root ZX-mini, Root ZX-II, and Sirona integrated apex locator successfully determined the location of the apical constriction and demonstrated high reliability. The use of lubricants did not influence the accuracy of these devices. However, significant differences were identified between the devices, with Root ZX-mini and Root ZX-II demonstrating superior performance compared to the Sirona integrated device.

## Figures and Tables

**Table 1:** Descriptive statistics of distance measurement to apical constriction with and without EDTA gel lubricant.

Device/Medium	Mean ±Std. Dev. (mm)	%95 Confidence Interval		*p*-Value
		Lower Limit	Upper Limit	
Root ZX mini DRY	0.456 ± 0.437 a	0.391	0.520	<0.0001
Root ZX mini EDTA	0.503 ± 0.449 a	0.437	0.569	
Root ZX II DRY	0.471 ± 0.453 a	0.404	0.537	
Root ZX II EDTA	0.484 ± 0.458 a	0.416	0.551	
Sirona Integrated DRY	0.704 ± 0.559 b	0.621	0.786	
Sirona Integrated EDTA	0.733 ± 0.501 b	0.659	0.807	

a,b Different letters indicate presence of statistical difference. Statistical significance set at *p* < 0.05.

**Table 2:** Two-way ANOVA table evaluating the significance of the effects of EDTA gel and EAL type, individually and combined, on measurement accuracy.

Source of Variance	Sum of Squares	df	Mean Square	F	Sig. *
Device	38.447	2	19.224	39.351	<0.0001
Medium	0.007	1	0.007	0.015	0.90
Device * Medium	0.362	2	0.181	0.371	0.69
Error	515.877	1056	0.489		
Total	578.307	1062			
Corrected Total	554.694	1061			

* Statistical significance set at *p* < 0.05.

## Data Availability

The data that support the findings of this study are available from the corresponding author upon reasonable request.
